# A Multifaceted Crisis: Sickle Cell Disease Complicated by Pulmonary Embolism, Avascular Necrosis, and Numb Chin Syndrome

**DOI:** 10.7759/cureus.85556

**Published:** 2025-06-08

**Authors:** Joudi Habbal, Amjad M Mohamadiyeh, Hussein W Khudhur, Waqar Gaba, Salah Abdelrahman

**Affiliations:** 1 Internal Medicine, Sheikh Khalifa Medical City, Abu Dhabi, ARE; 2 Clinical Sciences, University of Sharjah, Sharjah, ARE; 3 Radiology, Sheikh Khalifa Medical City, Abu Dhabi, ARE; 4 Neurology, Sheikh Khalifa Medical City, Abu Dhabi, ARE

**Keywords:** acute chest syndrome (acs), avascular necrosis, hbss, lower lip paresthesia, numb chin syndrome, pulmonary embolism, scd, sickle cell crisis, vaso-occlusive crisis (voc)

## Abstract

Sickle cell disease (SCD) is a multisystem disorder often leading to serious complications like avascular necrosis (AVN), pulmonary embolism (PE), and numb chin syndrome (NCS) during vaso-occlusive crises. While well-known complications such as stroke and acute chest syndrome are frequently encountered, rare manifestations, including NCS, often remain underdiagnosed. A 32-year-old male with SCD presented with severe lower back pain and dyspnea. On arrival, he had tachycardia and hypoxia, requiring oxygen support and extensive analgesic support. Laboratory results showed anemia, leukocytosis, and elevated D-dimer. CT pulmonary angiography (CTPA) confirmed segmental pulmonary embolism, while magnetic resonance imaging (MRI) of the hips demonstrated AVN of the bilateral femoral heads. During his hospital stay, the patient also developed gradual numbness of the chin and lower lip, raising concern for NCS. Although MRI of the head was inconclusive, the patient’s improvement after exchange transfusion suggested - but did not confirm - vaso-occlusive involvement as a potential cause. Treatment included analgesia, anticoagulation for PE, and exchange transfusion, resulting in symptomatic relief, especially for neurological symptoms. A multidisciplinary follow-up plan was arranged to ensure comprehensive care. SCD-related vaso-occlusion can lead to life-threatening complications, including PE and AVN, both of which were confirmed with imaging in this case. NCS is an important neurological manifestation in sickle cell crisis. Although imaging did not confirm NCS, the patient’s specific symptoms and response to exchange transfusion support the likelihood of vaso-occlusive involvement. This highlights the limitations of standard imaging in diagnosing NCS and suggests the need for further specialized imaging techniques, such as contrast-enhanced MRI of the mandible or PET scans, to confirm the diagnosis. This case underscores the importance of recognizing atypical manifestations in SCD, such as NCS, emphasizing the need for a multidisciplinary approach to manage complications like AVN, PE, and NCS, to prevent further morbidity.

## Introduction

Sickle cell disease is an inherited hemoglobinopathy where the hemoglobin becomes abnormal (hemoglobin S), causing red blood cells to become sickle-shaped, leading to recurrent vaso-occlusive crises and end-organ damage [1]. The epidemiology of sickle cell disease (SCD) reveals that it is most prevalent in Sub-Saharan Africa, with occurrences also found in some areas of the Mediterranean, the Middle East, and India, where frequencies can reach as high as 34% of the population. Case reports have described sickle cell disease (SCD) complications across a wide age range, from 11 to 60 years [2]. The presentation of sickle cell disease and its severity can vary significantly, from mild pain attacks to more serious, life-threatening conditions [3]. The pathophysiology of vaso-occlusive crises (VOC) includes microvascular obstruction, inflammation, and tissue ischemia, caused primarily by the entrapment of sickled erythrocytes and activated leukocytes in the microcirculation [3]. These same mechanisms contribute to complications such as pulmonary embolism (PE) and avascular necrosis (AVN). In pulmonary embolism (PE), chronic hemolysis, inflammation, and endothelial injury create a prothrombotic environment [4]. While in avascular necrosis (AVN), recurrent ischemic episodes impair perfusion to weight-bearing joints, leading to necrosis of the femoral heads [4]. Sickle cell disease (SCD) has been linked to various central nervous system disorders, primarily due to vascular phenomena [2].

Numb chin syndrome (NCS) is a rare sensory neurological complication of sickle cell disease (SCD) that affects the chin and lower lip. It typically presents as hypoesthesia, paraesthesia, or pain reported in a few cases, primarily resulting from damage to the inferior alveolar or mental nerve [2,5]. Though often associated with malignancy, only a few instances have reported the development of numb chin syndrome (NCS) associated with sickle cell crisis, highlighting the uncommon nature of this complication [2,6]. The true prevalence of NCS remains unknown, but it is likely underestimated due to underrecognition and underreporting [2]. Here, we present a case of a patient with sickle cell disease with a history of recurrent vaso-occlusive crises (VOC) who, upon this admission, developed pulmonary embolism, bilateral avascular necrosis of the hip joints, and numb chin syndrome (NCS).

## Case presentation

A 32-year-old male with sickle cell disease and a history of splenectomy presented to the emergency department with severe lower back pain and shortness of breath for three days. The pain was sharp, primarily localized to the sacral region, radiating to the right lower limb, and worsened with ambulation despite analgesia. His shortness of breath was persistent, exacerbated by exertion, and not associated with cough, hemoptysis, or orthopnea. A review of systems was negative for chest pain, fever, leg swelling, rash, or any other acute symptoms. His baseline functional status was fully independent, though he had recently been hospitalized for a similar episode of lower back pain, attributed to a vaso-occlusive crisis (VOC), precipitated mainly by non-compliance with hydroxyurea (500 mg, TID) and folic acid (5 mg, once daily) due to financial barriers. He was discharged on hydroxyurea (500 mg, TID), folic acid (5 mg, once daily), acetaminophen-codeine (500 mg - 30 mg, TID, for one week), and ibuprofen (400 mg, TID for three days only). Despite prior counseling, medication adherence remained poor due to financial constraints, contributing to symptom recurrence and readmission. His sickle cell history was otherwise uncomplicated, with no prior acute chest syndrome, stroke, or exchange transfusion. He underwent a splenectomy during childhood in his home country, though he does not recall the reason. On arrival, the patient appeared to be in moderate respiratory distress with vital signs as listed in Table 1.

**Table 1:** Patient vitals in the emergency department.

Vital signs	Values	Normal ranges
Heart rate	73 beats per minute	60-100 beats per minute
Oxygen saturation %	99% on 3L of oxygen via nasal cannula	95-100%
Respiration	28 breaths per minute	12-20 breaths per minute
Blood pressure	114/76 mmHg	Systolic: 90-120 mmHg; diastolic: 60-80 mmHg
Temperature tympanic	36.9°C	36.5°C-37.5°C

He was alert and oriented but in acute pain. Cardiovascular examination was regular with normal heart sounds. Pulmonary examination revealed decreased air entry on the right base of the chest, with no wheezing, crackles, or other added sounds on auscultation. Musculoskeletal examination revealed marked sacral tenderness with limited lumbar range of motion due to pain, particularly in flexion and extension. Hip examination demonstrated reduced internal and external rotation. A straight leg test was negative for radicular pain, which rules out nerve root compression. There were no visible deformities or joint swelling. The lower extremities showed no signs of acute swelling or erythema.

Initial laboratory investigations revealed microcytic anemia with a raised reticulocyte count. A peripheral blood smear demonstrated anisocytosis, microcytes, poikilocytosis, elliptocytes, sickle cells, and Howell-Jolly bodies. Hemoglobin electrophoresis confirmed a predominance of HbS (88.9%) with mildly elevated HbF (6.5%) and HbA2 (4.6%). Additionally, lactate dehydrogenase and inflammatory markers were elevated with regular renal and liver function tests. Troponin I was normal (Table 2).

**Table 2:** Initial laboratory results.

Test	Result	Reference range
Hematology		
Hemoglobin (Hb)	94 g/L	Male: 130-170 g/L
RBC count	3.97 × 10⁹/L	4.7-6.1 × 10⁹/L
WBC count	9.5 × 10⁹/L	4.0-11.0 × 10⁹/L
Platelet count	399 × 10⁹/L	150-400 × 10⁹/L
Mean corpuscular volume (MCV)	70 fL	80-100 fL
Mean corpuscular hemoglobin (MCH)	23.7 pg	27-33 pg
Red cell distribution width (RDW)	18.8%	11.5-14.5%
Reticulocyte count	6%	0.5-2.5%
Biochemistry		
Lactate dehydrogenase (LDH)	496 IU/L	125-220 IU/L
C-reactive protein (CRP)	28.7 mg/L	<5 mg/L
Creatinine	82 µmol/L	64-110 µmol/L
Aspartate aminotransferase (AST)	39 IU/L	10-40 IU/L
Alanine aminotransferase (ALT)	45 IU/L	7-56 IU/L
Total bilirubin	10.8 µmol/L	3-17 µmol/L
Direct bilirubin	4.7 µmol/L	<7 µmol/L
Albumin	37 g/L	35-50 g/L
Troponin I	5.120 ng/L	<12 ng/L
Hemoglobin analysis		
HbS	88.9%	-
HbF	6.5%	<1% in adults
HbA2	4.6%	2.5-3.5%

Electrolytes, including potassium, magnesium, sodium, calcium, and phosphorus, were all within normal ranges. Electrocardiography (ECG) showed normal sinus rhythm without ischemic changes or conduction abnormalities.

The patient was initiated on oxygen therapy via nasal cannula at 3 L/min in the emergency department to optimize oxygen delivery. Aggressive multimodal pain management was provided, including intravenous (IV) morphine 2.5 mg every 20 minutes as needed (PRN), IV ketorolac 30 mg (single dose), and IV paracetamol 1,000 mg, along with patient-controlled analgesia (PCA) morphine (2 mg per press) for additional pain relief. The patient was closely monitored for respiratory depression, sedation, and hypotension to ensure safe administration of analgesics. Additionally, the patient received a 1,000 mL IV bolus of normal saline (0.9%) to improve perfusion. Given the increased thrombotic risk in sickle cell disease, prophylactic low-molecular-weight heparin (LMWH) of 40 mg every 24 hours was initiated. The patient was admitted to a high dependency unit (HDU) as a case of sickle cell vaso-occlusive crisis for further pain control, close hemodynamic monitoring, and further evaluation of potential complications.

Within 48 hours, the patient continued to deteriorate, exhibiting worsening tachycardia (heart rate reaching 110 bpm) and tachypnea (respiratory rate reaching 40 breaths per minute). His oxygen requirements remained elevated, maintaining an oxygen saturation of 97% on 4 L/min via nasal cannula. Blood pressure remained stable at 124/84 mmHg. Given the clinical worsening and high thrombotic risk associated with sickle cell crisis, pulmonary embolism (PE) was strongly suspected.

Chest X-ray (CXR) showed atelectatic bands at the bilateral lung bases, small pleural effusions, and no pneumothorax. Arterial blood gas (ABG) revealed acute respiratory alkalosis with hypoxemia and a normal coagulation profile with elevated D-dimer (Table 3).

**Table 3:** Diagnostic workup - ABG and coagulation profile.

Additional laboratory findings		
Arterial blood gas (ABG)		
pH	7.5	7.35-7.45
pCO_2_	37.1 mmHg	35-45 mmHg
pO_2_	60 mmHg	80-100 mmHg
HCO_3_	29 mmol/L	22-28 mmol/L
Lactate	0.9 mmol/L	0.5-2.2 mmol/L
Coagulation profile		
Activated partial thromboplastin time (aPTT)	34.4 s	27.7-42.1 s
International normalized ratio (INR)	1.1	0.9-1.2
Prothrombin time (PT)	14 s	12-15 s
D-dimer	3.06 µ/mL	<0.5 µ/mL

**Figure 1:**
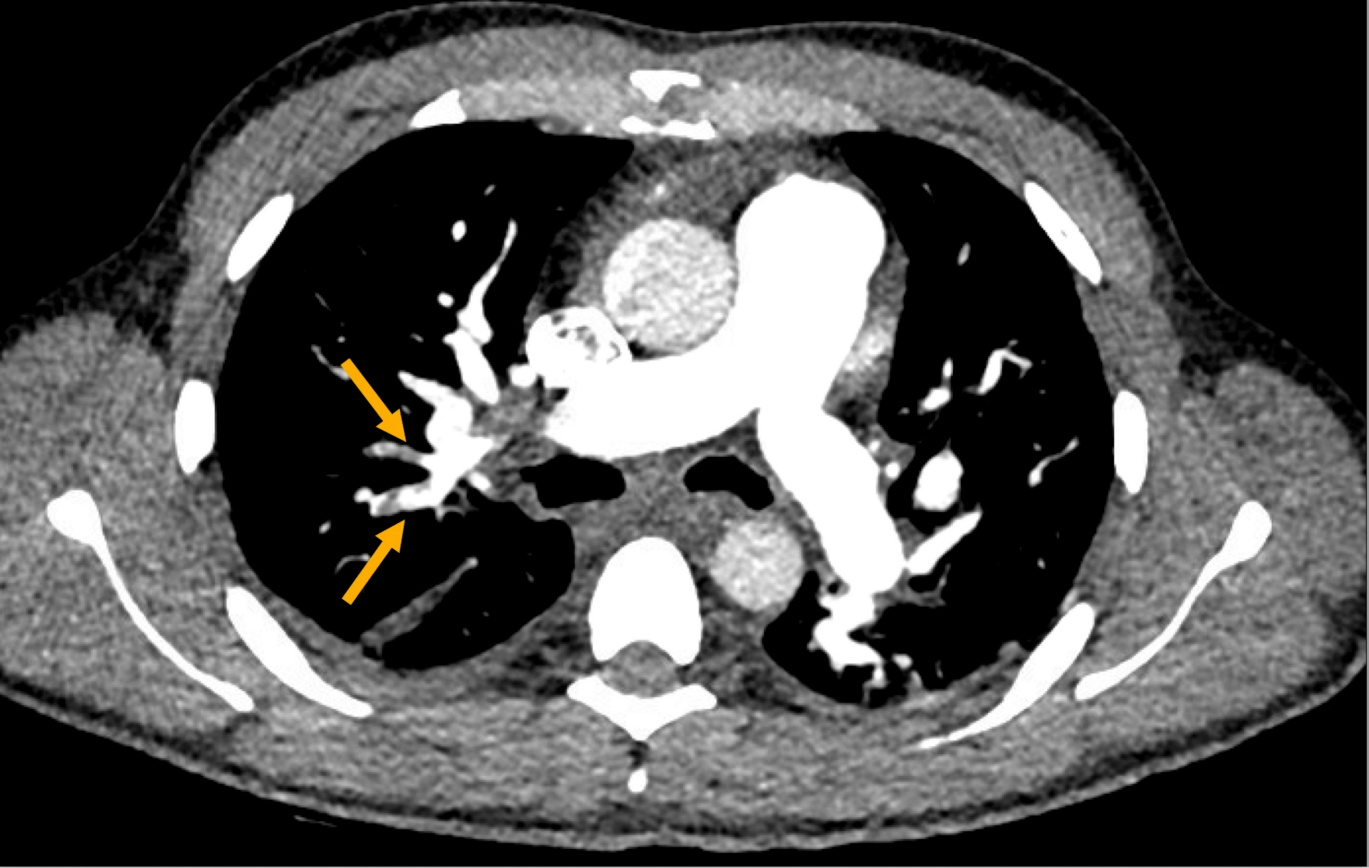
Contrast-enhanced CT pulmonary angiogram showing segmental pulmonary embolism. Axial CT image shows filling defects (arrows) in segmental branches of the right upper lobe pulmonary artery, consistent with acute embolism.

Computed tomography pulmonary angiography (CTPA) showed multiple filling defects, confirming right upper lobe segmental PE (Figure 1). Additionally, bilateral lower lobe consolidation/atelectasis and small bilateral pleural effusions were noted. The patient was initiated on therapeutic anticoagulation with enoxaparin (LMWH) at a dose of 1 mg/kg (68 mg) subcutaneously every 12 hours. He was also followed by the physiotherapy team for chest physiotherapy and was encouraged to use incentive spirometry at least five times daily to ensure adequate chest expansion and oxygenation. An echocardiogram assessed the potential cardiac sequelae from the pulmonary embolism. The ECHO demonstrated normal left ventricular size and ejection fraction of 55%, mild tricuspid regurgitation with normal right ventricular systolic pressure, and no significant structural or functional abnormalities.

**Figure 2:**
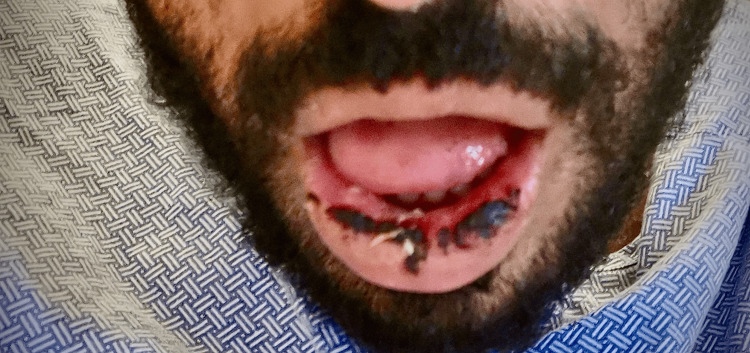
Clinical photograph: bruising of the lower lip. Photograph of the patient demonstrating ecchymosis of the lower lip, correlating with altered sensation in the mental nerve distribution.

Upon hospital stay day three, the patient began experiencing new-onset numbness over the lower third of his face, particularly in the chin area. His mother observed that he was unintentionally biting his lower lip during meals, leading to spontaneous bleeding and mucosal erosions (Figure 2).

Neurological examination revealed cranial nerve deficits isolated to the mandibular division of the trigeminal cranial nerve (CN V3), with intact sensation in the upper thirds of the face but hypoesthesia in the chin region. The remainder of the cranial nerve examination was unremarkable, with preserved facial symmetry, regular ocular movements, and no speech or swallowing difficulties. Motor and sensory examinations of the upper and lower limbs were regular, with no deficits in strength, reflexes, or coordination. Neurology consultation was obtained to evaluate potential central or peripheral causes of this perioral numbness. A non-contrast magnetic resonance imaging (MRI) of the brain and neck was performed, revealing no acute intracranial abnormalities, stroke, evidence of mass effect, bone lesions, or demyelination.

Additionally, a magnetic resonance angiogram (MRA) of the head and neck showed no significant vascular pathology. The neurologist concluded that the symptoms were most consistent with numb chin syndrome (NCS), a rare but recognized neurological manifestation of sickle cell disease. A magnetic resonance imaging (MRI) of the mandible would be the gold standard to diagnose numb chin syndrome (NCS); it was not performed in this case. Malignancy was considered in the differential but was deemed less likely given the absence of systemic signs or focal lesions on imaging. Therefore, in the context of ongoing vaso-occlusive crisis and absence of central pathology, the patient’s symptoms were attributed to numb chin syndrome secondary to sickle cell disease. Symptomatic management was initiated with pregabalin (75 mg, bedtime) and alpha-lipoic acid (600 mg, daily) for neuropathic pain relief. The patient was reassured that the numbness would resolve by improving the sickle cell crisis. Given the severity of the patient's condition, the hematology team recommended an urgent exchange transfusion with six units of red blood cells. By day four or five of admission, the patient showed a marked clinical improvement following the transfusion, with a resolution of the respiratory distress and the perioral numbness. Repeat labs showed HbS of 39.4%, and arterial blood gas (ABG) analysis confirmed adequate oxygenation without evidence of worsening respiratory compromise.

**Figure 3:**
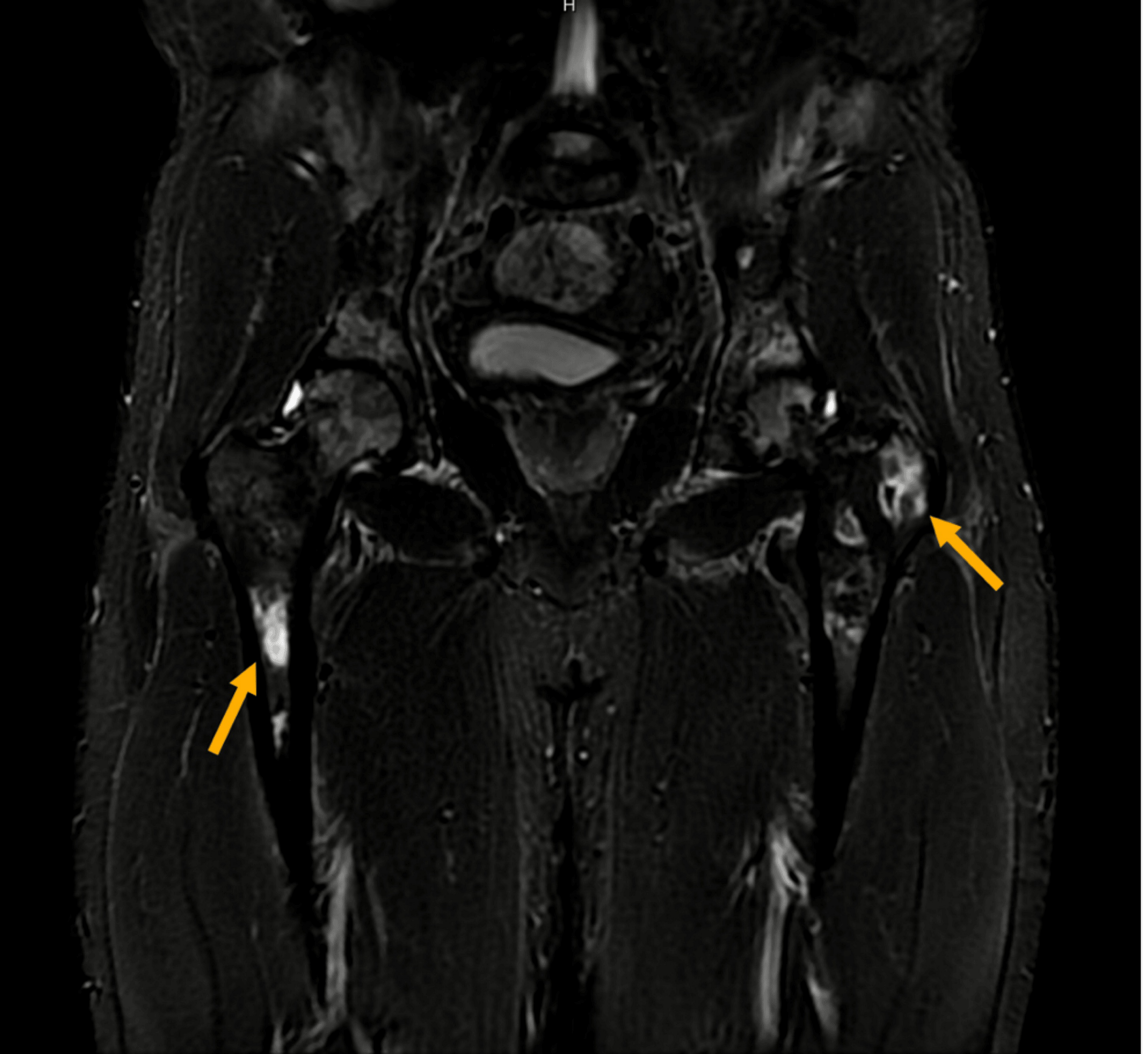
Coronal MRI demonstrating avascular necrosis of the femoral heads. Coronal short tau inversion recovery (STIR) MRI image shows bilateral femoral head hypointense areas (arrows), consistent with avascular necrosis.

Later during his hospital stay course, despite the improvement of vaso-occlusive (VOC) symptoms, the patient continued experiencing worsening bilateral hip pain and increasing difficulty with ambulation despite being on oral oxycodone 10 mg twice daily, intravenous (IV) oxycodone 10 mg every 6 hours as needed (PRN), and pregabalin 75 mg at bedtime. Given his persistent pain and functional decline, concern for avascular necrosis (AVN) was raised. A multi-sequence plain magnetic resonance imaging (MRI) of the hips was performed to evaluate for bone infarcts and joint damage. The MRI examination was positive for multiple areas of hyper-intensity along the femur bone (Figure 3).

A diagnosis of avascular necrosis was established. Pain management was escalated to oral oxycodone (20 mg twice daily), intravenous (IV) oxycodone 10 mg every 6 hours as needed (PRN), along with pregabalin (75 mg, bedtime). Moreover, the physiotherapy team was involved in rehabilitation strategies. On day nine of admission, the patient was deemed stable for discharge with an optimized pain management regimen and a comprehensive follow-up plan addressing his sickle cell disease complications, including avascular necrosis, pulmonary embolism, and numb chin syndrome attributed to the vaso-occlusive crises. His pain control was managed with oral acetaminophen (1,000 mg every six hours), extended-release oxycodone (20 mg twice daily), oxycodone (10 mg every six hours as needed), and pregabalin (75 mg at bedtime). Given his history of pulmonary embolism, therapeutic anticoagulation with edoxaban (30 mg daily) was initiated, with a planned six-month duration followed by reassessment, as this was his first provoked thromboembolic event. Additionally, he was continued on hydroxyurea (500 mg three times daily) and folic acid (5 mg daily) to reduce the frequency of vaso-occlusive crises (VOCs).

A multidisciplinary follow-up plan was arranged to ensure comprehensive care. The patient was referred to the hematology clinic to evaluate long-term disease management, including enrollment in an exchange transfusion program to prevent future vaso-occlusive crises (VOCs). An orthopedic surgery follow-up was scheduled to assess avascular necrosis (AVN) progression and determine the need for surgical intervention. Pain management and physiotherapy referrals were placed to optimize analgesia and improve mobility. Additionally, a neurology follow-up was arranged to monitor the resolution of his previously noted numb chin syndrome (NCS). The patient and his family were thoroughly counseled on the importance of medication adherence, including the risks and benefits of anticoagulation therapy. He was instructed to return to the emergency department if symptoms worsened.

With a structured outpatient plan emphasizing pain management, mobility preservation, and long-term hematologic monitoring, the patient was discharged in stable condition, with ongoing multidisciplinary support to prevent future complications of sickle cell disease.

**Table 4:** Summary of key complications and timeline. VOC: vaso-occlusive crises; SOB: shortness of breath; PE: pulmonary embolism; AVN: avascular necrosis

Hospital day	Events
0	Admission for VOC and SOB
2	Worsening respiratory status → PE diagnosed
3	Development of numb chin syndrome
4	Exchange transfusion performed
5	Chin numbness improved
6-7	New bilateral hip pain → MRI confirmed AVN
9	Discharged with follow-up plans

## Discussion

Vaso-occlusion and tissue ischemia are common complications of sickle cell disease (SCD) caused by the presence of abnormal hemoglobin S. This results from a point mutation in the beta-globin gene on chromosome 11, where glutamic acid is replaced by valine. The mutated hemoglobin causes red blood cells to adopt a sickle shape, impairing blood flow through small blood vessels and resulting in vascular obstruction, tissue ischemia, and hemolysis. Most of the cases reported were associated with genotype SS [2,4].

Numb chin syndrome (NCS) is a rare complication of sickle cell disease mainly caused by acute vaso-occlusive crisis or mandibular osteomyelitis [7]. The mental nerve, responsible for providing sensory innervation from the chin and lower lip, is anatomically predisposed to developing numb chin syndrome (NCS). It courses through the mandibular canal, making the terminal branch of the inferior alveolar nerve (mental nerve) susceptible to compression and ischemia when the canal is infiltrated, leading to numbness in the chin and lower lip [8].

Numb chin syndrome typically starts with unilaterally altered sensation, which can progress to bilateral mandibular symptoms. Patients often experience paresthesia or dysesthesia along the inferior alveolar nerve. Potential causes include metastatic mandibular lesions, trauma, cysts, inflammatory disorders, mandibular atrophy, dental procedures, neurological conditions, diabetes, sarcoidosis, amyloidosis, and sickle cell vaso-occlusion [9]. While numb chin syndrome in sickle cell disease likely reflects vaso-occlusion, malignancy (e.g., breast, prostate) was excluded given the lack of systemic symptoms and clinical suspicions [2,6].

A systematic review of 73 patients with numb chin syndrome revealed that the most commonly used imaging techniques were mandibular X-rays and head CT scans, followed by cerebral MRI [2]. CT scans provide greater detail than X-rays; however, both imaging modalities had similar findings in patients with numb chin syndrome (NCS). Nonetheless, CT scans are preferred over X-rays to evaluate for bone destruction or masses [10]. MRI is commonly used to assess brain parenchyma and abnormalities in the inferior alveolar nerve pathway in NCS cases. When requesting imaging, it is important to confirm that the mandibular region is included, as standard brain MRIs often exclude this area, potentially resulting in missed diagnoses [11].

The management of NCS in SCD remains unclear, with various treatment approaches reported in individual case studies aimed at addressing the underlying cause. Patients typically receive standard therapy for vaso-occlusive crises (VOC), including hydration, analgesia, and in more severe cases, blood transfusions [12]. For neuropathic pain, medications such as pregabalin or gabapentin may be considered for pain relief. Currently, there is limited data on the effectiveness of other treatments, such as antidepressants, antiepileptics, or neural blockades, in cases related to SCD [13].

## Conclusions

In conclusion, numb chin syndrome (NCS) in sickle cell disease (SCD) remains poorly understood due to limited evidence, leaving a lack of knowledge about its causes, progression, and treatment. A thorough evaluation by a neurologist and an SCD specialist, supported by a multidisciplinary team, is crucial. Imaging is essential to rule out other potential causes, such as local infections, primary neoplasms, or metastatic disease, and to visualize the trigeminal nerve in detail. Management primarily involves treating the underlying trigger and providing adequate pain relief. However, no clear guidelines have been established for treating patients with numb chin syndrome (NCS) in sickle cell disease (SCD).
